# Is the change of winter wheat yield under warming caused by shortened reproductive period?

**DOI:** 10.1002/ece3.403

**Published:** 2012-11-02

**Authors:** Ruixing Hou, Zhu Ouyang, Yunsheng Li, Glenn V Wilson, Hanxia Li

**Affiliations:** 1 Key Laboratory of Ecosystem Network Observation and Modeling, Institute of Geographic Sciences and Natural Resources Research, Chinese Academy of SciencesBeijing, 100101, China; 2 Yucheng Comprehensive Experiment Station, China Academy of ScienceBeijing, 100101, China; 3 USDA-ARS National Sedimentation LaboratoryOxford, Mississippi, 38655, USA

**Keywords:** Global warming, phenological stage, reproductive period, tiller, winter wheat, yield

## Abstract

Previous reports from laboratory-controlled experiments and models considered that a shorter reproductive period could be the main reason for wheat yield reduction in the warmer world. However, this conclusion needs to be proved carefully by field-scale experiments. In this study, a field-scale continuous open-warming experiment was conducted to quantify the adjustment of winter wheat growth and yield under conventional tillage (CT) and no-till (NT) systems in the North China Plain (NCP). Canopy temperatures were warmed using infrared heaters between 1.0 and 1.6°C (daytime and nighttime, respectively) above the control. Wheat yields under CT were not significantly reduced over the two seasons (2010 and 2011), but yields under NT were 3.3% and 6.1% lower, respectively. The growing seasons for both CT and NT were shortened 6 days in 2010 and 11 days in 2011; however, the reproductive periods were maintained. The shortened days were due to a significantly shorter springtime re-greening stage followed by minimal changes in other phenological stages (jointing, flag completed, heading, anthesis, and grain-filling). The temporal advance by warming resulted in lower growing-season mean air temperatures (MAT) for warmed plots than the control from 0.23 to 4.22°C for the same subsequent phenological stages. Warming increased the number of tillers m^−2^ and kernel weight, but tended to decrease the number of spikes m^−2^ in the two tillage systems. The heavier kernels offset the yield reduction from smaller number of spikes. Warming increased the wheat aboveground biomass from 10% to 20% suggesting the potential to sequester more CO_2_. This study suggests that winter wheat might adjust its growth (shortened vegetative period to maintain reproductive period) to partly compensate for the negative effects from global warming in this temperate irrigated cropland.

## Introduction

The Intergovernmental Panel on Climate Change (IPCC 2007) predicted an increase in mean ambient temperature between 1.8 and 5.8°C by the end of 21st century. As one of the most essential resources to world food supply, wheat yield is sensitive to temperature change (Schmidhuber and Tubiello 2007; Piao et al. 2010; Lobell et al. 2011). Given the complex physiological responses to climate change with uncertainties from several aspects (Tubiello et al. 2007), there are increasing concerns on this issue.

Change of phenology duration is an essential factor for wheat yield. Previous studies had found that warming will shorten wheat phenology duration and decrease wheat yield, mainly due to a shorter growing period, which decreases the duration of photosynthesis and wheat mass accumulation (Porter and Gawith 1999; Wheeler et al. 2000; Hatfield et al. 2011). The reduction would be 4–7% for each 1°C raised (Hatfield et al. 2011). Under climate change, grasses have the ability to acclimate to the temperature rise by adjusting their phenology (Sherry et al. 2007; Hovenden et al. 2008). Similarly, wheat might also adopt to climate change (Lavalle et al. 2009). White et al. (2011) observed earlier anthesis and maturity under warming plots than controls across 12 planting dates by an open-warming experiment in America, but minimal effect on wheat yield for winter planting dates. Zhang et al. (2010) observed that the date of wheat heading under 2.3°C warming was advanced 9 and 14 days in 2008 and 2009 growing seasons, respectively. However, it is not clear whether warming influences all phenological stages equally, or do vegetative and reproductive responses differ. Liu et al. (2009) found a shorter vegetative period, but stable response for reproductive period while the whole growing season was getting shorter from 1980 to 2005 in the North China Plain (NCP). Xiao et al. (2008) considered that wheat would benefit from advancement in anthesis and grain-filling periods. Therefore, greater knowledge of specific changes in phenological stages would help to understand the impacts of global warming on wheat yield.

Spikes per unit area, number of kernels per spike, and kernel weight are the determining components of wheat yield. However, there were contradictory reports about the impact of temperature elevation on wheat yield components between laboratory-controlled and field-scale-controlled experiments. For example, temperature rise significantly decreased kernel weight in laboratory studies (Chowdhury and Wardlaw 1978; Wheeler et al. 1996; Prasad et al. 2008), whereas field-scale warming studies found kernel weight would be increased (Patil et al. 2010; Zhang et al. 2010) or stable when planting wheat in winter (White et al. 2011). For the internal assimilation competition between kernel development and non-reproductive plant components, variances in kernel weight could be caused by many aspects of wheat components. However, other components of wheat yield or the probable changes in assimilation competition are not well studied under field-scale warming conditions.

Previous reports about the relationship between temperature rise and plant aboveground biomass were variable with negative (Aggarwal 2003; Peng et al. 2004; Lobell and Ortiz-Monasterio 2007), positive (Luo et al. 2001, 2009; Patil et al. 2010), or stable responses for winter-planted wheat (Ottman et al. 2012). Change in aboveground biomass under global warming occurs as plants establish a new balance between photosynthesis and plant respiration. Taking the huge amount of aboveground biomass of crops around the world into account, agroecosystems have great potential to affect carbon budgets between air and terrestrial ecosystems. Thus, it is imperative to conduct field-scale experiments to evaluate the feedback of terrestrial carbon cycling to global warming.

During the past decades, conservation tillage systems have spread around the world. Compared with conventional tillage (CT), no-tillage (NT) can reduce evaporation due to residue covering the soil surface and less soil disturbance. Under expected climate change, Ortiz et al. (2008) considered that NT could help wheat systems to adapt to climate change by better water-holding capacity and soil erosion prevention. The impact on wheat yield is not clear as NT has been found to increase, maintain, or decrease wheat yield (Nguyen and Dao 1989; Carr et al. 2003; Kumudini et al. 2008). In the present studied field, Hou et al. (2012) observed that wheat yield tended to be lower under NT than CT from 2004 to 2009. Based on the analysis of wheat yield components, Kumudini et al. (2008) considered that less tillers under CT could result in higher yield in CT. Wheat tillering could be affected by warming; there are reports that temperature rise decreases tillers per unit area (Batts et al. 1997; Prasad et al. 2008). These studies indicate that the present state of wheat growth or grain production under CT and NT systems could change in the future due to global warming, but there is limited knowledge on what changes to expect or what to do to adopt to such impacts.

The objectives of this study were to evaluate the relationship between the change of wheat yield and phenology stages under warming. Infrared heaters were used to conduct a field-scale open-warming experiment to examine the effects of warming on growth and yield response of winter wheat under NT and CT systems in an irrigated cropland in the NCP during 2010 to 2011. Infrared heaters have been recommended as an effective method to simulate global warming in field-scale experiments (Aronson and McNulty 2009). They have been used successfully in grassland ecosystems (Luo et al. 2001; Wan et al. 2009) and cropland ecosystems (Zhang et al. 2010; Wall et al. 2011).

## Materials and Methods

### Site description

**Figure 1:**
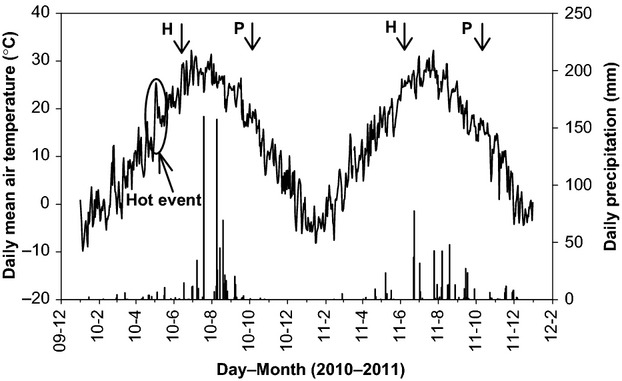
Daily mean air temperature (MAT) (line) and daily precipitation (solid bars) in 2010 and 2011. Data from weather station about 100 m from the study site. The MAT in the circle indicated a hot event in 2010. H and P stand for harvest and planting, respectively. The arrows with H and P indicate the harvest and planting dates of winter wheat in the unwarmed plots for no-till and conservation tillage treatments from 2010 to 2011.

This study was conducted on long-term (since 2003) conservation tillage fields at Yucheng Comprehensive Experiment Station of China Academy of Science (36°50′N,116°34′E,elevation is 20 m). These experimental fields were established as a bilateral project on conservation tillage between the USDA-ARS National Sedimentation Laboratory and the Institute of Geographic Sciences and Natural Resources Research of Chinese Academy of Sciences in the NCP. It is located in a temperate semi-arid climate, with annual mean temperature of 13.4°C and mean precipitation of 567 mm during the past 25 years (from 1985 to 2009). Approximately, 70% of annual precipitation occurs between June and September. For 2010 and 2011, mean temperatures were 13.3 and 12.8°C and precipitation amounts were 739 and 566 mm, respectively (Fig. 1). The soil is classified as a Calcaric fluvisols according to the FAO-Uneson system; surface soil texture is silt loam (sand, 12%; silt, 66%; clay, 22%), according to the USDA classification system. The surface soil pH is 8.6. Winter wheat (*Triticum aestivum L*.) and summer maize (*Zea Mays L*.) double cropping is predominant in the NCP. Depending on precipitation, winter wheat is irrigated using local groundwater.

Winter wheat was seeded on 6th and 7th October in 2010 and 2011, respectively, and harvested in early June. Winter wheat was irrigated two times each season in 2010 and 2011 from March to May (70–80 mm each time). For CT system, after maize harvest, standing stubble of each treatment was cut to about 10 cm for maize, and all other residues were removed. A rotary tiller was used with a tillage depth of about 10–15 cm, which fully incorporated standing stubble into the soil before winter wheat planting. For NT, maize residues were chopped into pieces (about 5-cm length) by hand and retained on the soil surface. The residue mass retained for NT was about 10 Mg ha^−1^ year^−1^ with 4 Mg ha^−1^ year^−1^ of wheat and 6 Mg ha^−1^ year^−1^ of maize.

In this experiment, the total N application rate for NT and CT treatments was 285 kg N ha^−1^ year^−1^ for wheat. Part or all of the total N, along with phosphorus (P) and potassium (K) was applied as compound fertilizer, which was an inorganic chemical fertilizer containing N (as urea), P (as P_2_O_5_), and K (as K_2_O) at the rate of 12:19:13 with application rates of 116 kg N ha^−1^, 178 kg P ha^−1^, and 122 kg K ha^−1^ each year. For CT system, the remaining 169 kg N ha^−1^ year^−1^ was applied as urea. For NT system, the rest of total N was 122 kg N ha^−1^ year^−1^ as a single urea application and the remaining 47 kg N ha^−1^ year^−1^ as maize residue with 0.8% N. All other management procedures were identical for the two systems with herbicide (2,4-d butylate) and insecticide (40% dimethoate) spraying in May.

### Experimental design and management

In this study, a complete random block design was used with warming as the primary factor and tillage system as the secondary factor based on the original NT and CT plots. Sixteen 2 × 2 m blocks, four treatments (CT with and without warming and NT with and without warming) replicated four times were arranged in a 4 × 4 matrix. There was a 5-m border between adjacent blocks and at least 10 m between the plots. The heated block in each pair was continuously warmed using MSR-2420 infrared heater (Kalglo Electronics Inc., Bethlehem, PA) since 4 February 2010. The infrared heater was suspended 3 m above ground, and did not generate any visible light to influence crop phenology (Sherry et al. 2007). The control (no-warming) blocks were the same shape and size with a “dummy” infrared heater suspended 3 m above ground to stimulate shading effects of the infrared heater.

### Measurement protocols

Soil temperature at 5-cm depth and soil moisture (0–10 cm) were monitored by PT 100 thermocouples and FDS100 soil moisture sensors (Unism Technologies Incorporated, Beijing, China). Two pairs of thermocouples and moisture sensors were arranged symmetrically and vertically to the infrared heater or “dummy” with 1-m distance between the pair in each plot and connected to a datalogger. Temperature and moisture measurements were taken every 10 min. Temperature of the canopy after anthesis was measured by a thermal imager (Model SC2000 Therma CAM; Flir Systems, Danderyd, Sweden) at 0900, 1500, and 2100 h each day from 19 April to 26 April in 2011. The wave band of the thermal imager was 8–14 μm. To correct for heater radiation and sky radiation reflected off the crop canopy, the longwave downward radiation of heater (*L*_DWR′_) was based on the formulas by Tian et al. (2008):


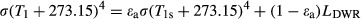
(1)


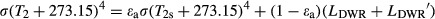
(2)

where σ is the Stefan-Boltzmann constant (5.67 × 10^−8^ W m^−2^ K^−4^), *L*_DWR_ and *L*_DWR′_ are the longwave downward radiation from the sky and the heater, respecitvely. *T*_1_ is the “cool” point temperature where canopy is not warmed by heater; *T*_1s_ is the surface temperature of the “cool” point. *T*_2_ is the “warm” point temperature where canopy was warmed by heater; *T*_2s_ is the surface temperature of the “warm” point. *T*_1_ and *T*_2_ were measured by an aluminum plate with an emissivity (ε_a_) of 0.15. *T*_2_ was measured on the canopy at different position from middle to side in one plot. *L*_DWR_ is the sky longwave downward radiation; data of *L*_DWR_ were obtained from a weather station near the study field every 30 min. *L*_DWR′_ was the longwave downward radiation of infrared heaters. Formulas (1) and (2) were used to obtain an average radiation of the infrared heater of 92 W m^−2^.


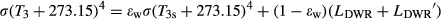


where *T*_3_ was the wheat canopy temperature measured by the thermal camera, and *T*_3s_ was the surface temperature of wheat canopy. Wheat emissivity (ε_w_) was assumed to be 0.98 (Tian et al. 2008). Mean air temperature (MAT) was recorded by a nearby weather station, which was about 100 m from the study field.

Winter wheat phenology stages were observed from re-greening to harvest. The date of a phenological stage was recorded when 50% of the winter wheat in the experiment plot had changed its developmental stage. Height of winter wheat was recorded based on the average value of 20 randomly chosen plants in each plot. Measurements were made from re-greening to anthesis (considering the different dates for re-greening and anthesis between warmed and unwarmed plots, the measurement days were based on unwarmed plots) every 7 and 5 days after 19 March 2010 and 15 March 2011, respectively. Aboveground biomass was sampled as two random groups (each had 20 winter wheat plants) in each plot at harvest, dried at 70°C for 48 h to constant weight and then weighed. Number of kernels, fertile spikelets, and sterile spikelets per spike of each group were counted in laboratory. Tillers (at anthesis) and spike density (at harvest maturity) were counted by hand. Grain yield was harvested in each plot with 2-m width and 2-m length after the grain was air-dried. Then, the kernel weight, based on 1000 kernels, was determined from each plot. The harvest index (HI) was calculated as the ratio of yield weight to total plant weight expressed as a percent.

### Statistical analysis

The effects of warming on microclimate, wheat growth, and components of wheat yield were determined by one-way analysis of covariance. Significance was determined by least significant difference at the 0.05 level using SPSS for Windows, version 11.5 (SPSS Inc., Champaign, IL). The duration of wheat phenological stage changes was determined by Sigmaplot 10.0 software (Systat Inc., Chicago, IL).

## Results

### Microclimate

Diurnal warming elevated mean daily soil temperature (T) at 5-cm depth by 1.09 and 1.60°C, respectively, for NT and CT systems from February 2010 to June 2011, Table 1. Also for the mean daytime and nighttime temperature, soil T elevation under CT (1.51 and 1.68°C) was higher than NT (0.73 and 1.34°C). However, the mean maximum and minimum T elevations were lower under CT (1.01 and 1.50°C) than NT (1.46 and 1.66°C). The impact of warming on the canopy temperature under two tillage systems was the same, Table 1; higher T during the night (1.62°C) and lower in the daytime (0.95°C). The other obvious impact of warming on soil between these tillage systems was soil moisture. Warming decreased the volumetric soil moisture at 0–10-cm depth by 3.8% (15.3 ± 0.5% vs. 19.1 ± 0.4%), but only 1.8% (18.6 ± 0.7% vs. 20.4 ± 1.3%) under NT.

**Table 1:** Changes due to warming treatment in mean soil and canopy temperature (T), and soil moisture (v/v%) for no-tillage (NT) and conventional tillage (CT) systems. Soil T and moisture were measured from February 2010 to July 2011. Canopy temperature was measured at the end of April 2011 for the two tillage systems

Treatments	NT	CT
Diurnal mean soil T (°C)	1.09 ± 0.14	1.60 ± 0.30
Daytime mean soil T (°C)	0.73 ± 0.17	1.51 ± 0.19
Nighttime mean soil T (°C)	1.34 ± 0.11	1.68 ± 0.20
Maximum soil T (°C)	1.46 ± 0.23	1.01 ± 0.14
Minimum soil T (°C)	1.66 ± 0.23	1.50 ± 0.19
Daytime mean canopy T (°C)	0.95 ± 0.19	
Nighttime mean canopy T (°C)	1.62 ± 0.13	
Mean soil moisture (v/v%)	−1.87	−3.84

All *P* < 0.05. The minus sign indicates a decrease.

### Impacts of warming on wheat growth

**Figure 2:**
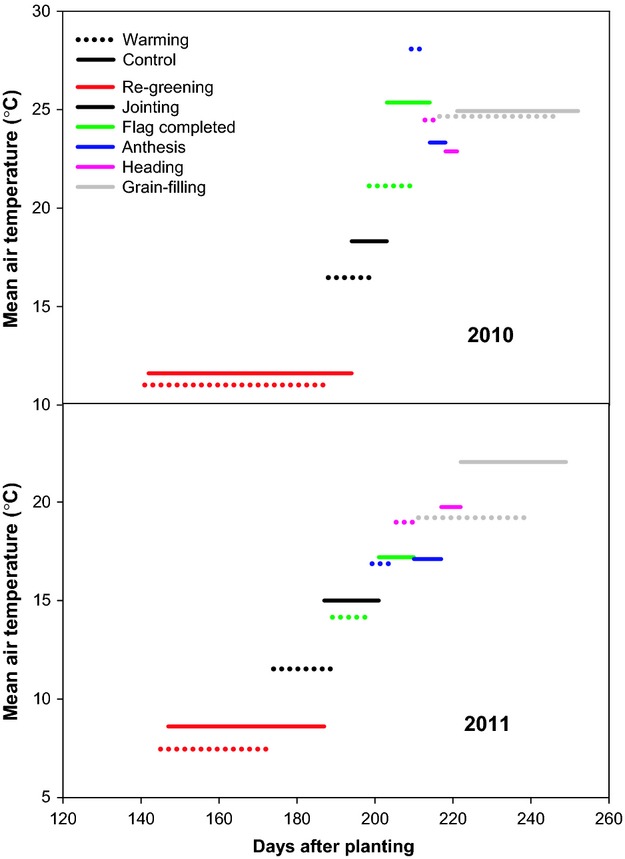
Durations of six phenological stages (R, re-greening; J, jointing; F, flag completed; H, heading; A, anthesis; G, grain-filling) from re-greening to maturity harvest and mean air temperature (MAT) for the six stages in 2010 (upper) and 2011 (bottom). Dotted lines stand for warming treatment; solid lines stand for control.

**Figure 3:**
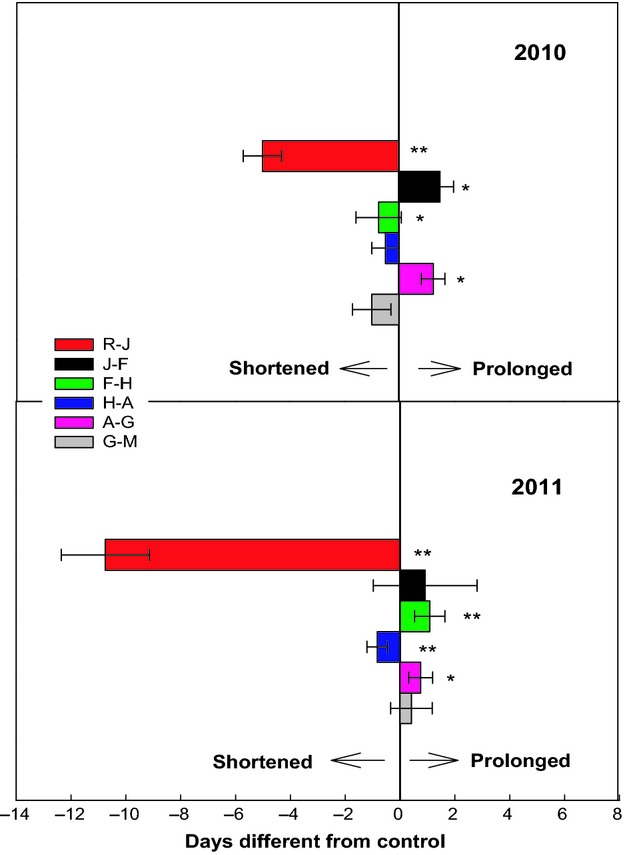
Change of days of six phenological stages from re-greening to harvest (R, re-greening; J, jointing; F, flag completed; H, heading; A, anthesis; G, grain-filling; M, maturity harvest) under warming compared with control in 2010 (upper) and 2011 (bottom). Data are mean ± standard error (SE) for the shortened or prolonged phenological stage durations. Significance indicated by: *P < 0.05, and **P < 0.001.

Temperature rise obviously shortened wheat growing season from re-greening to maturity in 2010 and 2011 by 6 and 11 days, respectively, as shown in Figure 2. To better understand the impacts of warming on wheat phenology, phenological period from re-greening to maturity was divided into six stages: re-greening, jointing, flag completed, heading, anthesis, and grain-filling. Re-greening was significantly shorter (at *P* < 0.001 level) under warming both years by 5.0 and 10.8 days, respectively, Figure 3. However, changes of the other five phenological stages were <2 days compared with the control, and the changes were not consistent in trend or significance. Durations of jointing, and anthesis were significantly prolonged in 2010. Flag completion and anthesis were significantly prolonged in 2011; however, flag completion was significantly shorter in 2010 and heading significantly shorter in 2011. Grain-filling stage was not significantly different from the control for the 2 years. The duration of each stage was similar between NT and CT for warmed and control plots.

Winter wheat growth was significantly affected by warming, and the trend was consistent with the advancement in jointing due to shorter time in re-greening stage under warming. Wheat grew faster under warming during re-greening, and this advantage was maintained until maturity in both 2010 and 2011. As a result of the advancement in phenological stages under warming, the control treatments reached the subsequent stages at a later time (Fig. 2). The MAT measured at the nearby weather station was lower under warming than control plots (range from 0.23 to 4.22°C) during subsequent stages, except from 1 to 5 of May in 2010 at heading and anthesis stages during an individual hot event. The average MAT over all stages from re-greening to maturity was 17.51 and 13.44°C in the warmed plots for 2010 and 2011, respectively, as compared with 17.94 and 14.89°C for the respective control plots. The 0.43 and 1.45°C lower MAT for warmed plots was due to these stages being reached earlier in the growing season.

**Figure 4:**
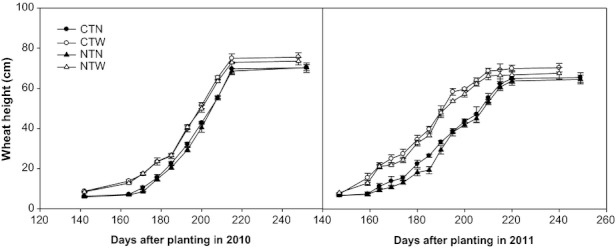
Wheat height from re-greening to harvest for the four treatments: conventional tillage with no warming (CTN); conventional tillage with warming (CTW); no-till with no warming (CTN); no-till with warming (NTW) in 2010 and 2011.

For CT and NT treatments, winter wheat height was significantly (*P* < 0.05) higher under warming than control and ranked in the same order in both years: CTW > NTW > CTN > NTN (Fig. 4). Warming not only accelerated the growth of winter wheat but also increased the accumulation in aboveground biomass. At harvest maturity, aboveground biomass was significantly (*P* < 0.05) increased by 10.0% and 19.6% under NT compared with the control in 2010 and 2011, respectively, and 13.4% and 16.8%, respectively, for CT.

### Impacts of warming on wheat yield and yield components

The effects of warming on wheat yield components and yield were not consistent under the two tillage systems. Differences in wheat yields were not significant at 95% level, Table 2, for either tillage system both years. However, wheat yields were numerically lower under warming than controls in NT systems by 3.3% and 6.1% in 2010 and 2011, respectively. In contrast, wheat yield under CT was numerically higher under warming than control by 1.6% in 2010, but equal in 2011.

**Table 2:** Wheat growth (number of tillers m^−2^ at jointing, increase in aboveground biomass [AGB] compared with the control), yield components (number of spikes m^−2^, number of fertile and sterile spikelets per spike, number of kernels per spike, and kernel weight), and harvest index (HI) over the two growing seasons (2010 and 2011)

Trts	No. of tillers m^−2^	No. of spikes m^−2^	No. of fertile spikelets spike^−1^	No. of sterile spikelets spike^−1^	No. of kernels spike^−1^	Kernel weight (mg)	Yield (Mg ha^−1^)	AGB (%)	HI (%)
2010									
NTN	916 (81)ab	489 (28)	16.1 (1.9)	1.4 (1.0)	35.3 (6.5)	36.6 (0.1)b	6.0 (0.3)bc	10.0 (4.1)	53.0
NTW	1046 (82)a	461 (25)	15.3 (2.3)	1.6 (0.8)	34.7 (7.4)	37.2 (0.1)a	5.8 (0.1)c		48.1
CTN	820 (93)b	510 (33)	15.6 (1.7)	1.0 (0.9)	34.6 (6.1)	36.2 (0.3)b	6.3 (0.2)ab	13.4 (5.2)	51.9
CTW	965 (79)ab	486 (29)	16.3 (1.8)	1.3 (1.0)	36.0 (4.8)	37.0 (0.2)a	6.4 (0.0)a		47.7
2011									
NTN	1063 (111)bc	511 (37)	15.0 (2.0)	1.7 (1.5)	34.6 (6.1)a	37.9 (0.0)b	6.6 (0.2)	19.6 (2.7)	60.2
NTW	1274 (86)a	483 (18)	14.7 (2.1)	2.7 (1.5)	32.1 (6.4)b	39.6 (0.8)a	6.2 (0.4)		48.7
CTN	926 (113)c	523 (24)	15.2 (2.2)	1.4 (1.1)	34.3 (7.0)a	37.2 (0.5)b	6.7 (0.2)	16.8 (4.0)	59.9
CTW	1150 (113)ab	490 (27)	16.2 (2.1)	1.3 (0.9)	35.0 (7.2)a	37.9 (0.3)b	6.6 (0.6)		51.7

Different letters in a column within a year designate significant differences (*P* < 0.05) among treatments.

Values are means with standard deviation in parenthesis (*n* = 4).

Treatments (Trts): conventional tillage without warming (CTN), conventional tillage with warming (CTW), no-till without warming (NTN), no-till with warming (NTW).

Tillers m^−2^ were increased 14.2% and 17.7% by warming in 2010 and 19.8% and 24.2% in 2011 for NT and CT systems, respectively. The difference between warming and control treatments was significant (*P* < 0.05) in 2011 (Table 2). Although not significantly different within tillage systems in 2010, the NT with warming had significantly more tillers than the CT without warming. Although not significant, warming appeared to reduce the number of spikes m^−2^ for both NT and CT systems; the mean decline was 28 spikes m^−2^ for both NT and CT systems in the two growing seasons. The kernel weight was significantly increased by warming for both NT and CT in 2010, but only for NT in 2011. The mean increase was 0.7 and 1.2 mg for CT in the 2010 and 2011 seasons, respectively. However, the effect of warming on other properties of the spikes, for example number of fertile and sterile spikelets and number of kernels per spike, was not consistent between tillage systems and years. Warming tended to decrease the number of fertile spikelets per spike by 0.8 and 0.3 in NT, but increase by 0.7 and 1.0 in CT for 2010 and 2011 season, respectively. The number of sterile spikelets per spike tended to increase in warming plots under NT both years, but the change was negligible and inconsistent in CT treatment. The grain numbers per spike tended to be reduced by 0.6 kernels spike^−1^ in 2010 and significantly (*P* < 0.05) reduced by 7.2% in 2011 under warming. Contrary to NT system, number of kernels per spike tended to increase under CT by 1.4 and 0.7 kernels spike^−1^ for 2010 and 2011, respectively, but these changes were not significant at the 95% level. Yield was either stable (CT) or slightly decreased (NT) and aboveground biomass increased due to warming. Thus, warming resulted in greater accumulation of biomass without an increase in yield. As a result, the HI decreased under warming. For the two tillage systems, HI decreased 4.9% and 11.5% for NT and 4.2% and 8.2% for CT in 2010 and 2011, respectively.

## Discussion

### Wheat yield and phenological stage duration

Previous laboratory-controlled (Sofield et al. 1977; Chowdhury and Wardlaw 1978; Prasad et al. 2008) and polyethylene-covered enclosure (Wheeler et al. 1996) studies have reported that wheat yield would be reduced and growth season shortened with a temperature rise. Hatfield et al. (2011) reviewed the reports and considered that the main reason for the reduction in wheat yield as temperature rises was the shortened growing season, which would decrease photosynthesis duration and limit assimilation. In contrast, Patil et al. (2010) found a negative relationship between soil temperature and the duration of wheat grain-filling, but the yield was stable. Another infrared field warming experiment on wheat found earlier anthesis and maturity across 12 planting dates (White et al. 2011), but yield was minimally changed for winter planting. In general, the lengths of crop phenological stages are determined by temperature as crops progress to the next growth stage when they accumulate enough heat units. A 25-year observation found that the duration of wheat post-flowering was stable while the pre-flowering duration was shortened under warmer environments in the North China Plain (Liu et al. 2009). This result is consistent with this study on changes in phenological stages, which suggest that warming promotes winter wheat to begin jointing earlier in spring, thereby reducing the re-greening duration, while other phenological periods were just advanced. Although the whole growing season was shortened, the minimal change in length of reproductive period (anthesis and grain-filling stages) should not be considered the main effects of warming for NT or CT systems in this region as the main advancement occurred due to shortening of time in re-greening.

Farooq et al. (2011) summarized the minimum, optimum, and maximum temperatures for different phenological phases in wheat. They found that wheat yield would decrease with MAT higher than the optimum temperature in anthesis and grain-filling stages. Asseng et al. (2011) suggested from model simulations that an average growing-season temperature increase by 2°C could cause 50% grain yield reduction in Australia because temperatures >34°C stimulate leaf senescence. However, the advancement in stages due to warming could result in the MAT being lower under warmed plots for the same stages as compared with the control, which reaches these stages at a later time. In this study, except for a hot event that occurred 1–5 May (shown in Fig. 1) at the anthesis stage of the warming treatment, the MAT under warming was 0.27°C lower in grain-filling stage in 2010, and 0.23 and 2.8°C lower for anthesis and grain-filling stages in 2011 than the controls. Xiao et al. (2008) studied the phenological stages and yield at low- and high-altitude sites, which were with a 2.2°C temperature difference, and also found that advanced anthesis moved the grain-filling period to a cooler and wetter period of the season, which could increase grain yield. This indicated that wheat may adjust its growth to compensate, at least partly, for negative effects of global warming in this temperate region.

The advancement by warming of the anthesis and grain-filling stages might help wheat to avoid heat stress and decrease the negative effect from *T*_max_ on yield. The MAT of phenological stages subsequent to re-greening under warming was lower than the imposed mean temperature rise and MAT of the control. Tao and Zhang (2012) predicted that wheat yield may benefit from maturing earlier, which could prevent it from severe high-temperature stress in the North China Plain. This study indicated that the elevated temperature by infrared heater was compensated partly by advancing of the reproductive phase, which thereby experienced decreased MAT.

### Wheat yield-formation and warming

In general, wheat yield is determined by the number of spikes per unit area, number of kernels per spike, and kernel weight. In this study, results were consistent with Donald's ideotype theories (Donald 1968), which postulated that the increase of competition for assimilation between developing kernels and non-reproductive plant components could result in tiller density increase. This means an increase in tillers would limit the assimilation availability for fertile tillers because of the increased competition between developing tillers and younger non-productive tillers. A soil warming experiment on wheat found that warming reduces the spike number m^−2^, but increases kernel weight, such that grain yield remains the same (Patil et al. 2010). It has been considered that the increase in assimilation competition between tiller and stem development could result in tiller or floret abortion (Thorne and Wood 1987; Slafer et al. 1999). Considering the non-significant change in the number of kernels per spike under the two tillage systems, the increased kernel weight could partly compensate for the negative effect of less fertile tillers, and help maintain the wheat yield. This was consistent with Darwinkel (1978) who also found that kernel weight increased as the number of spikes m^−2^ decreased. In another field-scale open-warming experiment on a wheat–rice system using infrared heaters, a 2.3°C temperature rise significantly increased the 1000 kernel weight from 1.94 to 4.6 g (Tian et al. 2011) and grain yield by 18.3% (Zhang et al. 2010) in China.

### Warming and tillage systems

Global warming likely will increase rates of evapotranspiration following a rain or irrigation. Therefore, soil moisture, one of the most important factors in crop growth and grain production, would be depleted more quickly. Conservation tillage is better in water-holding capacity relative to a conventional-tilled system (Lal 2009) due to mulch covering at least 30% of the surface. NT not only has even greater residue cover but also combined with the lack of soil disturbance; it generally results in increased infiltration. According to Ortiz et al. (2008), conservation tillage would help wheat to adapt to climate change due to soil erosion prevention and improved water retention. This study showed only a 2% decrease in soil moisture by volume for NT as compared with 4% decrease for CT system under warming as compared with the control. The small decrease in soil moisture under NT and CT by warming likely had a minimal impact on winter wheat growth and yield for such irrigated cropland. For wheat yield components under NT and CT systems, Kumudini et al. (2008) found that NT system increased internal competition for assimilates by increased tillering, and this resulted in wheat yield reduction relative to CT. In this study, warming increased tillers m^−2^ more in NT than CT (17.7% vs. 14.2% in 2010 and 19.8% vs. 24.2% in 2011), which might be one reason for yield reduction in NT, but minimum change in CT. Modhej et al. (2008) found that a reduction in the number of fertile spikelets and kernels per spike could result in wheat yield reduction, which supports the results of NT on the relationship between fertile spikelets and kernels per spike and yield. The opposite trend of the effects of warming on the number of fertile spikelets and kernels per spike between NT and CT could be another reason.

### Warming and wheat aboveground biomass

Changes in aboveground biomass accumulation by temperature increases would affect the contribution of agroecosystems to CO_2_ sequestration in a future warmer world. Lobell and Ortiz-Monasterio (2007) simulated the influence of elevated temperature on wheat biomass and found that daily minimum and maximum temperature increase by 1°C would decrease biomass 3–7% for three irrigated sites in western North America. Ottman et al. (2012) studied the effects of elevated temperature on 12 planting dates of wheat and found that wheat biomass tended to decrease under heating. The exception was winter planting, which exhibited a minimum change in the southwestern United States. In comparison, Luo et al. (2009) found that warming stimulates aboveground biomass by enhancing C4 dominance in grassland. Wan et al. (2009) found photosynthesis in warmed plots (night and diurnal warming) to be greater than respiration. They investigated this phenomenon and found it to be caused by photosynthetic overcompensation, which could result in carbon accumulation in the China steppe ecosystem. Patil et al. (2010) also found that *in situ* soil warming significantly increased aboveground biomass especially during anthesis. The increase in biomass in this study might be caused by greater photosynthesis rate during the shortened wheat growing period under warming and the photosynthesis rate also should be greater than respiration, but these need to be studied further. This phenomenon suggests that a future warmer world could have the potential to enhance carbon sequestration in agricultural ecosystem.

## Summary

In the temperate North China Plain, which is one of most important grain production regions of China, warming did not significantly affect wheat yield under either NT or CT systems. Although the period of re-greening was shortened, there were minimal changes in duration of other wheat stages, especially for the grain-filling stage. Stages subsequent to re-greening occurred sooner, and thus under cooler environments than for warming treatments. As a result, winter wheat might be able to adjust its growth and various yield components to minimize negative impacts from warming. For instance, tillering was increased by warming, which could negatively affect yield components, but kernel weight was also increased. The greater kernel weight compensated in part to prevent a yield reduction. Both NT and CT exhibited increased aboveground biomass accumulation. This increased C sequestration under warming could be an environmental benefit both for production of biofuels and reduction in atmospheric C levels.

Overall, the acclimatization of winter wheat growth to global warming could offset the negative response from temperature rise when considering food security in this temperate irrigated cropland. It may be important for the future to consider selection of varieties that reduce the stimulation of tillering by warming or improve other growth and yield components, especially for NT systems, for future environments.
